# Detection of active P-glycoprotein in systemic lupus erythematosus patients with poor disease control

**DOI:** 10.3892/etm.2012.667

**Published:** 2012-08-16

**Authors:** BO ZHANG, YING SHI, TIE-CHI LEI

**Affiliations:** Department of Dermatology, Renmin Hospital of Wuhan University, Wuhan, Hubei 430060, P.R. China

**Keywords:** P-glycoprotein, disease control, systemic lupus erythematosus disease activity index

## Abstract

Active P-glycoprotein (P-gp) molecules have been shown to transport steroids out of peripheral lymphocytes, resulting in poor responses to systemic steroid therapy in patients with systemic lupus erythematosus (SLE). This study was carried out to investigate the correlation between the expression or activity of P-gp in peripheral lymphocytes and disease control in SLE patients with a long history of systemic steroid treatment. A total of 60 SLE patients who had received systemic steroid treatment for longer than 6 months and 30 healthy subjects were monitored. SLE patients were subclassified into those with active and severely active forms of the disease according to their disease activity (estimated by SLEDAI-2000). The expression levels and activity of P-gp in peripheral blood lymphocytes were determined. Lymphocytes, obtained from three patients with severely active SLE, with high levels of P-gp expression were treated with cyclophosphamide, mycophenolic acid or emodin *in vitro* and Rh123-efflux activity was measured. P-gp expression in the peripheral lymphocytes of the SLE patients was significantly higher compared with that of the healthy controls, and a positive correlation between disease activity and P-gp expression levels was observed in these 60 patients. A significant increase in P-gp expression was observed in the severely active compared with the active SLE group. Treatment of lymphocytes with 100 μM cyclophosphamide or 100 μM emodin *in vitro* induced up to a 2-fold increase in the mean fluorescence intensity, as detected by the Rh123-efflux assay. In conclusion, the high expression levels of P-gp in the peripheral lymphocytes of SLE patients leads to poor disease control by systemic steroids. Emodin, an active ingredient derived from Chinese herbs, possesses a promising effect for overcoming P-gp-mediated steroid resistance by inhibiting the P-gp efflux function.

## Introduction

Systemic lupus erythematosus (SLE) is an autoimmune connective-tissue disease, predominantly affecting females aged between their late teens and early 40s, has a wide range of clinical features (1). Although SLE is prevalent worldwide, the Chinese population has a relatively high prevalence rate of 9–92 cases per 100,000 individuals (1,2). Prior to the addition of Benlysta as the most recent agent in 2011, there were few drugs for SLE treatment approved by the US Food and Drug Administration (FDA), including glucocorticoids, antimalarials and aspirin (3). Glucocorticoids are the main therapeutic strategy and the most effective anti-inflammatory drugs available for the treatment of a number of chronic autoimmune diseases, including SLE (4). However, when exhibiting poor responses, even to high-doses of systemic steroids, some SLE patients lose disease control, requiring other immunosuppressive therapies. Preventing steroid resistance and maintaining disease control are significant challenges to overcome in treating SLE patients.

The overexpression of P-glycoprotein (P-gp) in peripheral lymphocytes, which may lead to the exclusion of glucocorticoids, has emerged as one of the mechanisms involved in poor responses to steroid treatment in SLE patients (5). P-gp is a 170-kDa product of the multidrug resistance 1 (MDR-1) gene, a member of the ATP binding cassette transporter superfamily. P-gp molecules function as drug efflux pumps that transport numerous drugs out of cells, including antibiotics and cytotoxins, as well as several drugs commonly used for treating autoimmune diseases. This means that high expression levels of active P-gp in peripheral lymphocytes result in poor disease control by steroid therapy (6). Therefore, in the present study, the P-gp expression and activity of peripheral blood lymphocytes obtained from SLE patients who had previously undergone long-term steroid treatment was examined. Furthermore, the effects of P-gp inhibitors on lymphocytes from steroid-resistant SLE patients *in vitro* were investigated.

## Materials and methods

### Subjects

The Ethics Committee of Remin Hospital of Wuhan University approved this study and informed consent was obtained from all the healthy subjects and patients. The study included 60 SLE patients and 30 healthy subjects. All the SLE patients met the diagnostic criteria for SLE established by the American Rheumatism Association in 1997 and had received systemic steroids as the only systemic immunosuppressive therapy for ≥6 months (7). The disease activity of the SLE patients was assessed using the SLE Disease Activity Index-2000 (SLEDAI-2000), and all of the patients had the active form of the disease (SLEDAI-2000>4) (8–10). The SLE patients were further subclassified into an active SLE group, whose SLEDAI was ≤12, and a severely active SLE group, whose SLEDAI was >12 (10,11). The SLEDAI index is a global score index developed for the assessment of SLE disease activity, the range of which is between 0 and 105 (8,9). The index has been demonstrated to be reliable, to have construct validity and to be sensitive to change (8,9). Table I shows the demographic characteristics of the SLE patients and healthy controls.

### Isolation of peripheral blood lymphocytes

Peripheral blood lymphocytes were isolated by density-gradient centrifugation. Heparinized venous peripheral blood was obtained from the SLE patients and healthy controls. The blood was diluted by adding an equal volume of 0.9% NaCl. A total of 6 ml of diluted blood was carefully layered over 3 ml of lymphocyte separation medium (MP Biomedicals LLC. Solon, OH, USA) and was centrifuged at 800 x g for 20 min at room temperature in a swing-out rotor. After centrifugation, the mononuclear cells formed a distinct band at the sample interface. The harvested fraction was diluted with buffered RPMI-1640 medium (Invitrogen, Gaithersburg, MD, USA) to reduce the density of the solution, and the cells were pelleted by centrifugation for 10 min at 250 x g. Platelets were removed by layering the cells suspended in buffered RPMI-1640 medium and centrifuging them for 15 min at 350 x g. The pellets were used as mononuclear cells.

### P-gp expression assay

P-gp expression levels in lymphocytes were analyzed using standard flow cytometry procedures as described previously (12). Lymphocytes (1×10^6^) were incubated with 5 μl PE-conjugated anti-P-gp (CD243) monoclonal antibody (eBioscience, Inc., San Diego, CA, USA) or 5 μl PE-conjugated matched-isotype control antibody (IgG2a, eBioscience) for 30 min at 4°C, washed twice in PBS and subsequently analyzed on a FACScan (Becton-Dickinson, Mountain View, CA, USA). At least 10,000 cells were counted and lymphocytes were analyzed and separated according to their forward and side scatter characteristics. Results are expressed as the percentage of positive cells.

### Rhodamine 123 (Rh123) efflux assay

P-gp activity was measured using the Rh123 efflux assay as described previously (12). The fluorescent dye Rh123 (Sigma-Aldrich, St. Louis, MO, USA) was added to 2 ml lymphocytes (1x10^6^/ml) at a final concentration of 10 μg/ml and cells were incubated at 37°C for 30 min. Subsequently, the Rh123-loaded cells were washed twice with cold PBS and resuspended in PBS at the original volume. The Rh123-loaded cells were then separated into two aliquots, which were incubated for 30 min at 37°C in the absence or presence of verapamil (Sigma), a P-gp inhibitor (final concentration, 10 μM). Lymphocytes were kept on ice until they were analyzed using a FACScan (Becton-Dickinson). P-gp activity was determined for each subpopulation of cells by the percentage of the mean Rh123 fluorescence intensity (MFI) in the absence or presence of verapamil, i.e., P-gp activity (%) = [(MFI of cells with verapamil - MFI of cells without verapamil)/MFI of cells with verapamil] x 100.

### Reverse transcription-polymerase chain reaction (RT-PCR)

Total RNA was extracted from peripheral blood lymphocytes (5×10^6^ cells) of SLE patients and of healthy controls using TRIzol reagent (Invitrogen, Eugene, OR, USA) according to the manufacturer’s instructions and was quantified by measuring the absorbance at 260 nm. cDNA was synthesized from each total RNA using a Moloney murine leukemia virus reverse transcriptase first strand kit (Invitrogen, Shanghai, China). An aliquot of the RT product was then processed for DNA amplification by PCR using the primers: 5′-CCC ATC ATT GCA ATA GCA GG-3′ (sense) and 5′-GTT CAA ACT TCT GCT CCT GA-3′ (antisense) for P-gp/MDR-1; 5′-AGC GAG CAT CCC CCA AAG TT-3′ (sense) and 5′-GGG CAC GAA GGC TCA TCA TT-3′ (antisense) for β-actin. The reaction mixture (20 μl) contained 3 μl template cDNA, 2 μl of primers (50 pmol each of upstream and downstream primers), 5 μl RNase-free water and 10 μl 2X Es Taq MasterMix (including Es Taq DNA polymerase, 2X Es Taq PCR Buffer, 3 mM MgCl_2_ and 400 μM dNTP mix; CWBIO, Beijing, China). Thermal cycling conditions were as follows: denaturation at 94°C for 20 sec, annealing at 55°C for 30 sec, extension at 72°C for 30 sec for 25 cycles and a final extension at 72°C for 2 min. PCR products were separated on 2% agarose gels containing Goldview dye (SBS Genetech Co., Ltd., Shanghai, China). A 100-bp DNA Ladder (CWBIO) was electrophoresed on the same gel to determine product size. The gel was photographed and the amount of MDR1 and β-actin from each sample was analyzed by scanning densitometry. To normalize the results, the amount of P-gp/MDR1 was divided by the amount of β-actin.

### Treatment of lymphocytes with P-gp inhibitors in vitro

The lymphocytes were purified from three SLE patients with high levels of P-gp expression. Fluorescent dye Rh123 was added to 2 ml lymphocytes (1×10^6^/ml) at a final concentration of 10 μg/ml, and cells were incubated at 37°C for 30 min. Subsequently, the Rh123-loaded cells were washed twice with cold PBS and resuspended in PBS at the original volume. The Rh123-loaded cells were then separated into four aliquots, which were incubated for 30 min at 37°C in the absence or presence of 100 μM cyclophosphamide (Sigma), 100 μM mycophenolic acid (Sigma) or 100 μM emodin (Sino-FDA, Beijing, China). The cells were washed with cold PBS and resuspended in PBS, then kept on ice until they were analyzed using a FACScan.

### Statistical analysis

Data were shown as the median or mean ± SD. Differences between groups were determined using the non-parametric Mann-Whitney U test or Student’s t-test. Correlations between two variables were analyzed using Spearman’s rank correlation analysis. P<0.05 was considered to indicate a statistically significant difference. Statistical analyses were performed using SPSS 13.0 or GraphPad Prism version 5 (GraphPad Software, San Diego, CA, USA).

## Results

### Expression and activity of P-gp protein in peripheral blood lymphocytes from SLE patients

**Figure 1:**
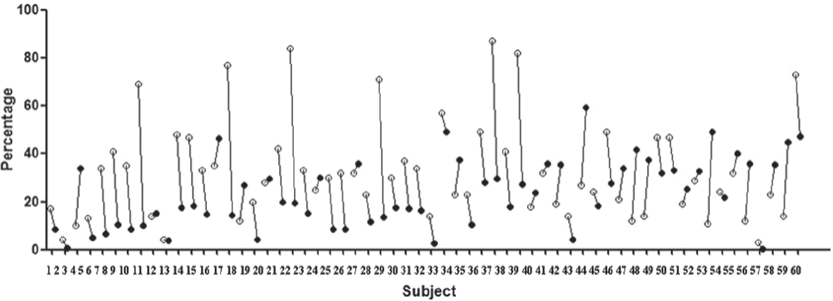
Expression and activity of P-gp in lymphocytes from 60 SLE patients. • represents a positive percentage of P-gp expression in the lymphocytes; ○ represents the efflux activity of P-gp in lymphocytes. P-gp, P-glycoprotein; SLE, systemic lupus erythematosus.

**Figure 2:**
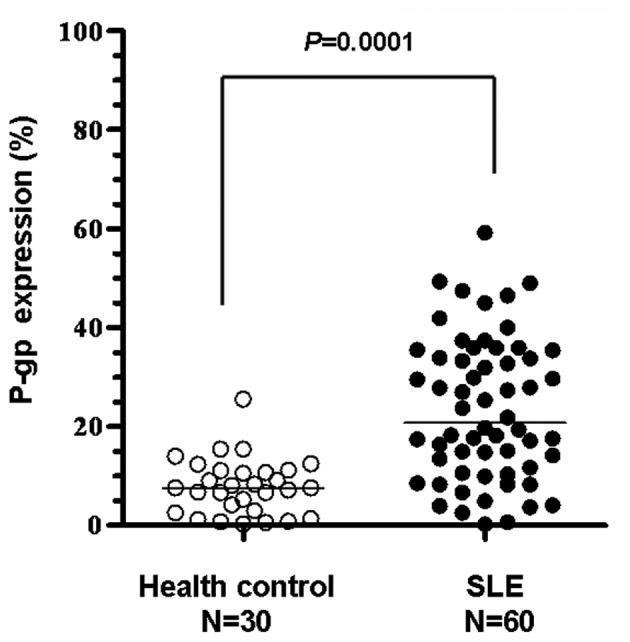
P-gp expression in the lymphocytes of SLE patients and healthy controls. P-gp expression levels of lymphocytes were detected using flow cytometry, as stated in Materials and methods. The differences between the two groups were analyzed using the Mann-Whitney U test and P<0.05 was considered to indicate statistically significant differences. The horizontal bars show the arithmetic median of each group. P-gp, P-glycoprotein; SLE, systemic lupus erythematosus.

A total of 60 SLE patients and 30 healthy volunteers were screened. We tested P-gp expression in lymphocytes using an anti-human MDR1 antibody and tested P-gp activity using an Rh123 efflux assay and the results were expressed as the percentage of positive cells or Rh123-efflux percentage, respectively. The majority of SLE patients expressed the P-gp protein in peripheral blood lymphocytes and the efflux-function of this P-gp was active in the majority of patients (Fig. 1). The expression levels of P-gp in the lymphocytes of healthy volunteers were minimal (Fig. 2) and P-gp expression in SLE patients with a long history of steroid use was significantly higher compared with the healthy controls (P=0.0001; Fig. 2).

### Differences in the activity and expression of P-gp in peripheral blood lymphocytes between patients with active and severely active SLE

**Figure 3:**
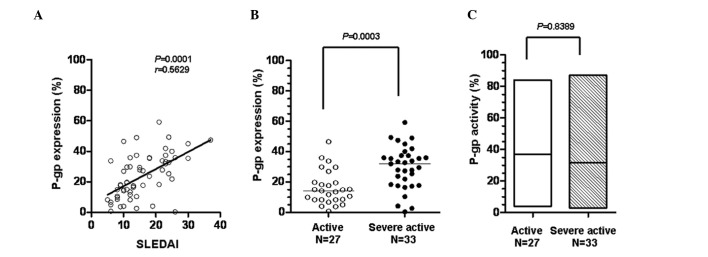
Expression and activity profiles of P-gp in lymphocytes from SLE patients with varying disease activities. (A) Correlation between P-gp expression levels and disease activity in SLE patients. (B) A significant difference in P-gp expression was observed between patients with active and those with severely active SLE. (C) No significant difference in P-gp activity was observed between patients with active and those with severely active SLE. The differences between the groups were analyzed using the Mann-Whitney U test and correlations between two variables were analyzed using Spearman’s rank correlation analysis. P<0.05 was considered to indicate a statistically significant difference. The arithmetic median is indicated with horizontal bars. P-gp, P-glycoprotein; SLE, systemic lupus erythematosus.

The SLE disease activity was estimated using the SLEDAI scoring system. To explore whether activity and expression of P-gp affect disease control by systemic steroids, the correlation of the SLEDAI scores with the P-gp expression levels of the 60 SLE patients was analyzed. The SLEDAI scores demonstrated a statistically significant positive correlation with the expression level of P-gp (P= 0.0001; Fig. 3A). The criterion for defining active SLE was SLEDAI≤12, otherwise SLE was defined as severely active. Fig. 3B shows that P-gp expression in the severely active SLE group was significantly higher than that in the active SLE group (P=0.0003), although P-gp activity was not significantly different between the two groups (P= 0.8389; Fig. 3C). These results suggest that high levels of P-gp expression in the peripheral lymphocytes of steroid-treated SLE patients may be the cause of poor disease control in SLE patients who have received long-term steroid treatment.

### Effects of intensive intravenous cyclophosphamide (CTX) administration on the expression and function of P-gp in peripheral bood lymphocytes in vivo

**Figure 4:**
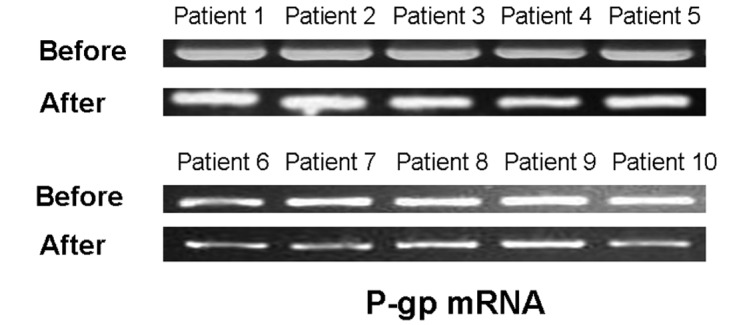
Effects of intensive intravenous CTX administration on P-gp mRNA expression in the lymphocytes of SLE patients *in vivo*. P-gp mRNA was tested by RT-PCR. The differences between groups were analyzed using the Student’s t-test. P<0.05 was considered to indicate a statistically significant difference. CTX, cyclophosphamide, P-gp, P-glycoprotein; SLE, systemic lupus erythematosus.

We aimed to determine whether P-gp expression in peripheral lymphocytes was inhibited by other intensive immunosuppressive therapies *in vivo*. We selected 10 SLE patients whose disease was highly active after >6 months of steroid therapy and placed them on an intravenous CTX regimen for the first time. Table II shows the demographic characteristics and therapeutic regimen of these patients, while Fig. 4 shows P-gp mRNA expression in the peripheral lymphocytes prior to and after CTX treatment. Of the 10 patients, 9 demonstrated no clear changes in P-gp protein expression and function. A marked reduction in P-gp expression was observed in only 1 patient (23.7 to 10.4%; Table II). None of the patients exhibited significantly different P-gp mRNA expression in their peripheral lymphocytes following CTX therapy (Fig. 4). In contrast to expectations, the results indicated that intensive intravenous CTX administration was unable to influence the P-gp expression in the peripheral lymphocytes of SLE patients *in vivo*.

### Effects of P-gp inhibitors on the efflux-function of P-gp in the peripheral lymphocytes of SLE patients in vitro

**Figure 5:**
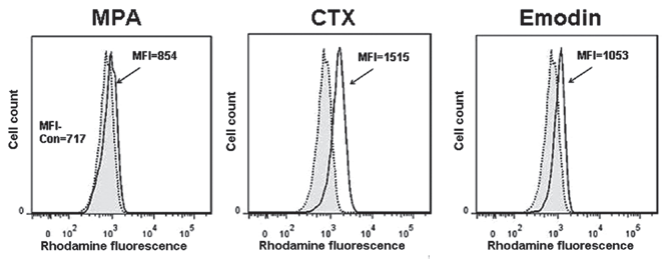
Effects of P-gp inhibitors on the efflux-function of P-gp in lymphocytes from steroid-resistant SLE patients *in vitro*. P-gp activity in lymphocytes from patients with SLE was measured using the Rh123-efflux assay, as stated in Materials and methods. The MFI was measured by flow cytometry in cells treated with 100 μM CTX, 100 μM emodin or 100 μM MPA. P-gp, P-glycoprotein; SLE, systemic lupus erythematosus; Rh123, Rhodamine 123; MFI, mean fluorescence intensity; MPA, mycophenolic acid.

In light of our previous findings, we designed an *in vitro* experiment to elucidate whether there are P-gp inhibitors, such as CTX, mycophenolic acid (MPA) and emodin, which affect the efflux-function of P-gp in the lymphocytes of SLE patients (13–15) Three patients with active SLE and high expression levels of active P-gp in their peripheral lymphocytes were selected. The Rh123-efflux assay indicated that 1.5- to 2-fold increases of the mean fluorescence intensity (MFI) occurred in cells treated with 100 μM CTX or 100 μM emodin, but no shift of the MFI peak was observed in cells treated with 100 μM MPA (Fig. 5). These results suggest that CTX and emodin has the potential to increase steroid accumulation in the lymphocytes of glucocorticoid-resistant SLE patients by inhibiting P-gp efflux activity.

## Discussion

SLE is an autoimmune disease characterized by an excess of autoantibodies produced by activated B cells and auto-reactive T cells. Glucocorticoids are key drugs for treating patients with active SLE. Insensitivity or resistance to systemic glucocorticoids in the treatment of active SLE patients has been reported and studied for a number of years. Several distinct mechanisms contributing to inhibit glucocorticoid activity in SLE patients have been identified, and overexpressed P-gp in peripheral lymphocytes was demonstrated to be one of these mechanisms (5,16–20). P-gp is a 170-kDa product of the MDR-1 gene, and functions as an energy-dependent trans-membrane efflux pump. P-gp is usually expressed in a wide variety of healthy tissues and cells, including the epithelial cells of the intestine, hepatocytes, the adrenal glands, renal proximal tubules and the endothelium of blood-brain and maternal-fetal barriers, as well as lymphocytes (6,21). The physiological role of P-gp has been defined as the detoxification and transport of metabolites due to its function as a one-way energy-dependent pump (6). Highly expressed P-gp in lymphocytes has been demonstrated to be involved in mechanisms of glucocorticoid insensitivity or resistance in several immune diseases besides SLE, including asthma, inflammatory bowel disease, immune thrombocytopenia and rheumatoid arthritis (RA) (16,22–26). However, little was known about P-gp in SLE patients. Assuming that the overexpression of active P-gp molecules caused the efflux of steroids from the intracellular space and thus prevented disease control in SLE patients by steroid therapy, we tested P-gp activity and expression in lymphocytes from SLE patients. Active and highly expressed P-gp was observed in the majority of SLE patients. Our findings indicate that for SLE patients with a long history of steroid use, P-gp expression level was positively correlated with disease activity and that the P-gp expression level in SLE patients influenced the level of disease control by long-term steroid administration.

Subsequently, we aimed to determine whether the expression or function of P-gp in the peripheral lymphocytes of SLE patients could be altered by a CTX regimen *in vivo*. Following treatment with 2.0 g intense CTX, only one patient demonstrated a notable decrease in the expression of P-gp protein although the patient’s P-gp mRNA levels did not change significantly as a result of CTX therapy. Our results also demonstrated that the immunosuppressive agent CTX was unable to suppress or promote P-gp expression. Three P-gp inhibitors, CTX, MPA (a metabolic product of mycophenolate mofetil) and emodin were then used in an attempt to block the efflux activity of P-gp (13–15), and CTX and emodin demonstrated characteristics of a competitor of the P-gp efflux activity.

In conclusion, we have demonstrated that the overexpression of P-gp in peripheral lymphocytes causes a poorer response to steroid therapy in the long-term and results in poor disease control in SLE patients. Emodin, an active ingredient derived from the herb, Chinese rhubarb (*Rheum palmatum*), possesses a promising effect for overcoming P-gp-mediated acquired steroid resistance.

## Figures and Tables

**Table I:** Clinical characteristics of the study patients.

Characteristic	Healthy control (n=30)	SLE patients (n=60)
Age, mean ± SD, years	30.5±9.4	29.3±10.5
Gender (female/male)	24/6	54/6
SLEDAI score, median (range)	-	14 (3–37)
SLE involvement, no. of patients		
Lupus nephritis	-	44
Blood abnormalities	-	30
Vasculitis	-	23
Arthritis	-	22
Myositis	-	9
Serositis	-	7
CNS lupus	-	1
Prednisolone (or equivalent) treatment		
Dosage, median (range), mg/day	-	22 (5–100)
Duration, median (range), months	-	26 (1–228)

SLE, systemic lupus erythematosus; SLEDAI, SLE disease activity index; CNS, central nervous system.

**Table II:** Effects of intensive intravenous cyclophosphamide administration on the expression and activiy of P-glycoprotein in peripheral bood lymphocytes *in vivo*.

Patients	Gender	Age (years)	Intravenous CTX dosage (g)	SLEDAI		P-gp expression (%)		P-gp activity (%)	
				Before therapy	After therapy	Before therapy	After therapy	Before therapy	After therapy
1	Female	19	2	37	37	51.2	47.4	73	69
2	Female	22	2	16	18	37.9	35.9	32	45
3	Female	17	2	23	23	41.9	37.4	14	33
4	Female	29	2	25	21	21	18.3	47	54
5	Female	32	2	30	11	35.5	29.4	84	72
6	Female	35	2	18	14	23.7	10.4	23	37
7	Male	20	2	23	23	35.9	32.8	34	44
8	Female	22	2	23	18	27	24.6	41	49
9	Female	24	2	19	19	27.9	23.9	57	44
10	Female	27	2	14	13	19.5	16.4	41	36

CTX, cyclophosphamide; P-gp, P-glycoprotein; SLEDAI, SLE disease activity index; SLE, systemic lupus erythematosus.

## Abbreviations:

SLE: systemic lupus erythematosus
MDR-1: multidrug resistance-1
P-gp: P-glycoprotein
SLEDAI: SLE Disease Activity Index
MFI: mean fluorescence intensity
Rh123: Rhodamine 123
CTX: cyclophosphamide
MPA: mycophenolic acid
Emodin: 1,3,8-trihydroxy-6-methylanthraquinone
